# Optimised concentration and purification of retroviruses using membrane chromatography

**DOI:** 10.1016/j.chroma.2014.03.023

**Published:** 2014-05-02

**Authors:** D.J. McNally, D. Darling, F. Farzaneh, P.R. Levison, N.K.H. Slater

**Affiliations:** a Department of Chemical Engineering and Biotechnology, New Museums Site, Pembroke St, Cambridge CB2 3RA, UK; b King's College London, 123 Coldharbour Lane, London SE5 9NU, UK; c Pall Europe Limited, 5 Harbourgate Business Park, Southampton Road, Portsmouth PO6 4BQ, Hampshire, UK

**Keywords:** Chromatography, Gene therapy, Virus, Membrane adsorption, Retrovirus

## Abstract

The ability of an anion exchange membrane to purify a γ-retrovirus was assessed and optimised with respect to different loading and wash buffers. Recoveries of infectious virus greater than 50% were consistently obtained, while specific titre was increased up to one thousand fold when compared to the material loaded. Specific proteins removed and retained by this optimised process were identified by mass spectrometry. It was possible to successfully bind and elute the equivalent of 1.27 × 10^8^ Ifu/ml of ion exchange membrane. This could then be highly concentrated, with infectious virus concentrated to a maximum of 420-fold compared to the load.

## Highlights

• An in investigation in to the use of membrane chromatography for the purification of a γ-retrovirus was undertaken.
• The first report of a capacity for γ-retrovirus binding to a membrane chromatography device is presented.
• A process that produces a large increase in concentration and purity of the studied γ-retrovirus was identified.
• Proteomic techniques were used to identify the protein impurities removed and co-purified with the virus containing eluate.

## 1 Introduction:

Retroviral gene therapy vectors are a promising class of gene delivery vectors and have been used in recent successful and ongoing clinical trials. They are advantageous in their ability to stably integrate genetic information into a target cell, carry a relatively large genetic payload and have low immunogenicity [1]. Globally, as of June 2013 there are 69 phase III clinical trials involving gene therapy treatments of all types and 266 trials at all stages involving retroviral vectors. These trials have a broad range of target diseases, from hereditary conditions such as x-linked severe combined immunodeficiency (X-SCID) to cancer [2]. Retroviral vectors used in gene therapy must be of high purity, high concentration and free of replication competent virus. Current methods of production are generally limited in scalability; thus, there exists an urgent need for the development of a production and purification process that can generate batches of vector with high yield and of sufficient quality for clinical use [3]. Concentration of a retroviral vector during downstream processing allows a reduction in the burden on processes downstream [4] and improvement in transduction efficiency [5].

Macroporous chromatography adsorbents such as monoliths, membranes and microcapillary films have demonstrated their ability to be used in virus purification [6–9]. More specifically, macroporous ion exchange membranes have demonstrated high dynamic capacity for viruses and other large biomolecules such as plasmid DNA [8,10]. This large dynamic capacity is attributed to their large pores, which allow high rates of mass transfer of large biomolecules to binding sites throughout the chromatographic media relatively independent of residence time [11]. Ion exchange membranes have high dynamic capacity for lentiviral vectors and an ability to substantially concentrate them. Both the Mustang^®^ Q and LentiSELECT anion exchange membranes have enabled successful concentration and purification of lentiviral vectors [8,9,12,13]. While no data on concentration factors achieved were reported by Kutner et al. [8], it is estimated that using a Mustang Q membrane with a volume of 0.18 ml they were able to concentrate a lentiviral vector an estimated 140-fold. This is significantly higher than the concentration factors achieved with any retrovirus by traditional chromatography, with a maximum concentration of between 1.5 and 5-fold being achieved by Rodrigues et al. [14] using a packed bed column while other membrane chromatography devices only achieved a maximum of 11-fold concentration [9], see Table 1.

Mustang Q membrane is a polyethersulfone (PES)-based membrane with a 0.8 micron nominal pore size and a surface coating of an irreversibly cross-linked polymer containing pendant Q groups [15].

These data indicate the utility of membrane chromatography for lentiviral vector purification. However, there is limited information available on the purification of γ-retroviral vectors, a frequently used retroviral gene therapy vector [2,9]. With concentration of viral gene therapy vectors being so important in their dosing and efficacy choosing a purification strategy that provides both a high concentration and satisfactory purification is vital. This paper examines the utility of the Mustang Q membrane for purification and concentration of retroviral vectors, with an emphasis on a γ-retroviral vector based on a murine leukaemia virus (MLV).

## 2 Materials and methods

### 2.1 Chemicals

The following were purchased from Sigma-Aldrich (Poole, UK): Sodium Hydroxide, Hydrochloric Acid, Sodium Chloride, Ammonium Acetate, Ethanol, Bovine RNase, Trypan Blue, Bovine Serum Albumin, Dithiothreitol (DTT), Tris–HCl, methanol, glycine, Tween-20 and acetone. All chemicals used were Molecular Biology grade.

### 2.2 Tissue culture reagents

Penicillin streptomycin solution, DMEM (Dulbecco's Modified Eagles Medium), Phosphate Buffered Saline (PBS), polybrene (hexadimethrine bromide), l-glutamine and Trypsin were purchased from Sigma-Aldrich (Poole, UK). RPMI (Rothwell Park Memorial Institute) and foetal calf serum (FCS) were purchased from Invitrogen (Paisley, UK). T75 and T175 tissue culture flasks and 96 well culture plates were obtained from Fisher Scientific (Loughborough, UK) and Star Labs (Milton Keynes, UK). FACS tubes were obtained from Bio-Rad (Hertfordshire, UK).

### 2.3 Cell lines

All cell lines were kindly supplied by Dr. David Darling of Kings College London. These include the EcoPack2 cell line for GFP carrying MLV retroviral vector production (Clontech, Saint-Germain-en-Laye, France) and the murine cell line 32Dp210 used for retrovirus titration.

### 2.4 Cell culture

All cell culture was performed at 37 °C with a 5% (v/v) CO_2_ enriched atmosphere, passaged at 80% confluency and counted using a haemocytometer. All culture media contained 10% (v/v) FCS and 100 U/ml penicillin and 100 mg/ml streptomycin.

### 2.5 Production of ecotrophic MLV retroviral vector

The EcoPack2 cell line (Clonetech, France) was cultured in T175 tissue culture flasks seeded at 1 × 10^5^ cell/ml until 80% confluence was achieved. At this point the supernatant was harvested and either used immediately or aliquoted and frozen at −80 °C.

### 2.6 Purification using the Mustang Q membrane

The Mustang Q membrane coin (0.35 ml membrane volume) and coin holder (Pall Europe, Portsmouth UK) were attached to a Masterflex^®^ L/S^®^ peristaltic pump (Cole-Palmer, London UK) equipped with a Masterflex^®^ L/S^®^ Easy-Load® pump head (Cole-Palmer, London, UK). MLV containing viral supernatant was produced as described above. The viral supernatant was then clarified using a 0.45 μm pore filter (Millipore, Elze, Germany) and titrated to 25 mM Tris–HCl pH 7 ± 0.1 or pH 8 ± 0.1 as required, using 1 M NaOH, with either no additional salt, 0.3 M NaCl, 0.6 M NaCl or 0.8 M NaCl, pH was determined using a sterile pH probe (Applisens ltd, NL). The Mustang Q coin assembly was sanitised at a flow rate of 3.5 ml/min using 10 ml of 1 M NaOH followed by 10 ml of 1 M NaCl and then conditioned using wash buffer containing the same NaCl concentration and at the same pH as the load or 800 mM NaCl pH 8 ± 0.1.One hundred fifty millilitres of titrated supernatant was then loaded onto the Q membrane. The Q membrane was then washed with 12 ml of wash buffer and eluted using 3.6 ml of 1.3 M NaCl, 25 mM Tris–HCl pH 8 ± 0.1. Fractions of 0.6 ml were collected and analysed for viral titre using FACS and total protein as described above. One experiment was performed using a Mustang Q XT Acrodisc^®^, all volumes were altered to account for the 0.85 ml Q membrane volume. All experiments were performed at room temperature, which was maintained between 18 and 21 °C.

### 2.7 Gradient elution experiments

150 ml of MLV containing viral supernatant was loaded onto the Mustang Q coin unit as described above. The MLV was then eluted using a step gradient from 0.9 M NaCl to 1.4 M NaCl 25 mM Tris–HCl pH 8 ± 0.1. Each step in the gradient consisted of 6 ml of elution buffer and was collected in 1 ml fractions, which were analysed for infective viral titre using FACS.

### 2.8 Large scale experiments using the Mustang Q coin unit

The Q membrane and viral supernatant were prepared as described above in larger volumes, up to 1000 ml. These were loaded onto the Q membrane at an initial flow rate of 10 membrane volumes a minute, during which the material that flowed through the Q membrane was collected.

### 2.9 Establishing infective viral titre using flow cytometry

This was performed using murine 32Dp210 cells plated in RPMI, 10% FCS, 1% P/S and polybrene at 4.4 μg/ml as in [6]. A Becton Dickinson FACScan was used to read the GFP fluorescence. The linear range of target cell GFP fluorescence to infective titre is between approximately 5 and 30%.

### 2.10 Total protein determination

Quantitative protein determination was carried out using a standard Bradford assay, (Sigma, Dorset UK). It was performed using 96 well plates in an EL340 plate reader from Bio Tek instruments (Bedfordshire, UK).

### 2.11 SDS-PAGE and silver stain analysis of protein content

All SDS-PAGE gels used were Invitrogen (Paisley, UK) 15 or 10 well 4-to-12% bis tris precast Nu-PAGE mini gels and were run according to the manufacturer's instructions. Invitrogen's Mark12™ unstained standard was used as a molecular weight marker at a 1 in 20 dilution. After running at 200 V for 35 min the gels were silver stained with the SilverSnap 2 stain kit from Pierce (Northumberland, UK). Reduction of samples was performed with DTT. Gels were photographed on a MEDALight light panel (Morco, UK).

### 2.12 Western blotting

A semi dry protocol was used, with the primary antibody being a rabbit polyclonal to MLV GAG from Abcam (Cambridge, UK) diluted 1 in 1500 in 5% skimmed milk powder (Marvel, Dublin, Ireland) in PBST (PBS containing 0.1% Tween-20). The secondary was an anti-rabbit IgG conjugated to HRP from GE Healthcare (Buckinghamshire, UK) diluted 1 in 1000. After the SDS-PAGE was run with Novex sharp pre-stained protein standards (Invitrogen, UK) the protein was transferred to PVDF membrane (GE Healthcare, Buckinghamshire, UK) using a Transfer-Blot Semi-Dry Transfer Cell (Bio-Rad, Hertfordshire, UK) at 15 V for 1 h. The blot was developed using the ECL Prime kit from GE Healthcare (Buckinghamshire, UK). Visualisation and imaging was performed using a G:Box system (Syngene, Cambridge, UK).

### 2.13 Preparation of samples for 2D-DiGE and mass spectrometry

Two dimensional difference gel electrophoresis (2D-DiGE) and electrospray ion trap mass spectrometry (ESI-Trap) followed by searching of the NCIB protein database was performed by the Cambridge Centre for Proteomics (CCP). To prepare samples to undergo these processes insoluble material had to be removed. Therefore, the samples were purified using ammonium acetate and acetone precipitation.

### 2.14 Measurement of p30 protein concentration

The measurement of retroviral p30 concentration was performed using the QuickTiter™ MuLV Core Antigen ELISA Kit (MuLV p30) (Cambridge Bioscience, Cambridge, UK) as directed. Each sample was performed in duplicate and read using an EnVision 2104 Multilabel reader (PerkinElmer, Massachusetts, USA).

### 2.15 Measurement of dsDNA concentration

dsDNA concentration was measured using the PicoGreen Assay kit from Invitrogen (Paisley, UK) according to the improved method proposed by Charlton and colleagues [16] using a Perkin Elmer LS50B plate reader (Massachusetts, USA).

## 3 Results and discussion

To establish the capacity of the Mustang Q membrane for an MLV based γ-retrovirus large volumes of MLV containing supernatants were prepared and loaded onto Mustang Q membrane devices. The supernatant that passed through the Q membrane was assayed for infective virus and viral capsid p30 protein content, where indicated. The Q membranes were eluted and the fractions collected were titred for infectivity.

**Fig. 1:**
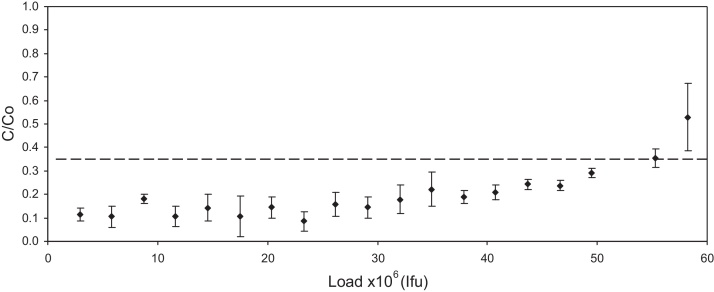
is a scatter graph showing the ratio of the measured infectious titre in the flow through relative to the load during an experiment where 1000 ml of murine leukaemia virus containing supernatant adjusted to 25 mM Tris–HCl pH 8 was loaded on to a 0.35 ml Mustang Q coin unit at 10 Membrane volumes a minutes at an initial flow rate of 3.5 ml/min with an average flow rate of 2.9 ml/min over the experiment. The load contained 7.86 × 10^7^ ± 3.36 × 10^5^ Ifu. Dashed line represent the limit of accurate quantification of the assay ≥5% GFP positive target cells. Infectivity was measured in triplicate.

Fig. 1 demonstrates the high capacity for MLV of the Mustang Q membrane. It shows the ratio of infectious titre in the flow through relative to infectious titre in the feed at different time points during the loading of 1000 ml of MLV supernatant onto a Mustang Q membrane. It can also be seen that the infective titre in the flow through is either below, or on the limit of accurate quantification. There is a gradual increase in infectivity towards the end of the load, suggesting breakthrough is occurring. A summary of three experiments in which large volumes of supernatant were applied to the Mustang Q coin assembly is shown in Table 2. In all three experiments it can be estimated that at least 70% of the loaded virus bound to the Q membrane with between 45 and 56% being recovered in the eluate. Macroporous adsorbers including the Mustang Q membrane have been shown to be able to adsorb large entities such as viruses independent of flow rate [8,11,13]. Therefore increasing the residence time of the load in the Mustang Q membrane would most likely not lead to increased recovery. However, a reduction in the total load below the 7.86 × 10^7^ ± 3.36 × 10^5^ Ifu used to produce Fig. 1 could increase recovery by reducing the amount of virus lost due to breakthrough.

**Fig. 2:**
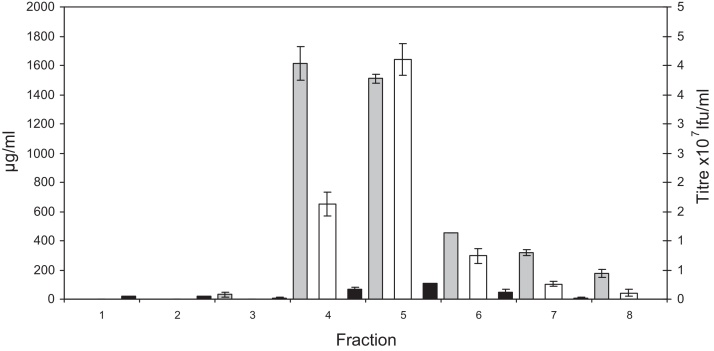
is a bar graph showing the titre, protein content and dsDNA content measured in elution fractions collected following the loading of 790 ml of murine leukaemia virus containing cell culture supernatant titrated to 25 mM Tris–HCl pH 8 onto a Mustang Q coin unit. Each fraction is of 0.6 ml. ■ is dsDNA, □ is Ifu ■ is protein.

Despite detection of infectious virus flowing through the membrane, the lack of clear evidence for significant breakthrough in the flow through fractions of these experiments demonstrates that anion exchange membranes have a large capacity for γ-retroviruses. The Q membrane was able to adsorb virus from a feed equivalent to over 2857 membrane volumes. This large capacity coupled with the high mass transfer rate of viruses within the Mustang Q membrane resulted in a high concentration of infective virus upon elution. Table 2 shows that the recovered virus was concentrated between 125 and 132-fold, with peak concentration factors of 420-fold, which represented 60% of the total recovered virus while 95% of the recovered virus was concentrated 221-fold. This is clearly demonstrated in Fig. 2 where the difference in the concentration of virus in the elution fractions can be seen, with fraction 5 containing 60% of the recovered virus in 600 μl.

**Fig. 3:**
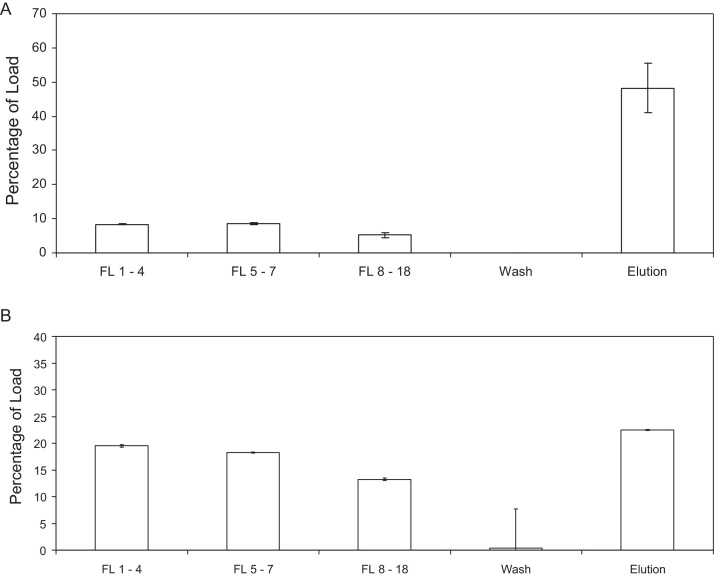
(A) is a bar graph showing the percentage of total loaded infectious virus detected in different fractions of an experiment where 1775 ml of murine leukaemia virus (MLV) containing supernatant adjusted to 300 mM NaCl 25 mM Tris–HCl pH 7 was loaded on to a Mustang Q XT Acrodisc at an initial flow rate of 10 membrane volumes a minutes, equal to 8.6 ml/min, with an average flow rate of 6.45 ml/min. The load contained 2.39 × 10^8^ ± 3.77 × 10^5^ Ifu. Flow through (FL) fractions 1–4 represents the first 650 ml of the flow through. FL 5–7 represents the subsequent 600 ml and FL 8–18 represents the final 525 ml. The titre in these fractions is an estimate as although infectivity was detected it is below the limit of accurate quantification of the assay ≥5% GFP positive target cells. ‘Wash’ represents a 20 ml wash with 300 mM NaCl 25 mM Tris–HCl pH 7 and ‘Elution’ represents elution from the membrane using 12 ml 1.3 M NaCl 25 mM Tris–HCl pH 8. (B) is a bar graph showing the percentage of total loaded MLV core p30 protein detected in different fractions, measured using a sandwich ELISA in duplicate.

To further investigate the capacity of the Mustang Q membrane 1775 ml of MLV containing supernatant was loaded onto a Mustang Q XT Acrodisc, a scale-down tool for the larger Mustang XT units. Loading commenced at an initial flow rate of 10 MV/min (8.6 ml/min), which by the end of the load had fallen to ∼6 ml/min. The fractions collected were analysed for both infective virus and MLV p30 capsid protein, the results of which can be seen in Fig. 3 and Table 3. As in the previous experiments some virus is detected in the flow through, but at a level too low to accurately quantify. As can be seen in Fig. 3A and B and Table 3 the measured or estimated viral titre at each section of the purification run does not match the viral protein concentration. The eluate contains 48.29 ± 7.18% of the loaded virus yet only contains 22.47 ± 0.01% of the loaded p30 protein. This equates to 1.29 Ifu to every 1 ng of p30 in the load and 307 Ifu to every 1 ng of p30 in the eluate. Therefore, while the p30 protein content cannot help indicate infectious titre it does show that viral protein not associated with infectious virus is removed in the flow through. Additionally, the mass balance only accounts for 74% of the viral protein present in the feed and indicates that there is viral protein bound to the membrane after elution, something previously observed by Grein et al. [17] during their study of baculovirus purification. On elution infectious virus was recovered concentrated 179-fold, with 99% of recovery found in two fractions concentrated 266-fold and the peak fraction, which contained 77% of the recovered virus being concentrated 410-fold. This peak concentration factor is similar to that observed with the Mustang Q coin.

The ability to provide large concentration factors whilst simultaneously providing extensive purification of a γ-retrovirus compares favourably with previously published data. Table 1 shows a selection of devices used for retrovirus concentration and the results obtained. The concentration factors presented here are almost double the highest estimated concentration factor achieved for any retroviruses, using any type of chromatography device. The previous maximum concentration factor achieved for a γ-retrovirus using chromatography is 11 fold, here we demonstrate concentration factors an order of magnitude higher [9]. The concentration factors achieved are also the highest recorded for this type of retrovirus, as well as being over 40 times higher than those reported with any packed bed chromatography device. Similar levels of concentration have only been demonstrated previously using ultrafiltration or a scaled down version of the Mustang Q membrane [8,9,14,18,19].

**Fig. 4:**
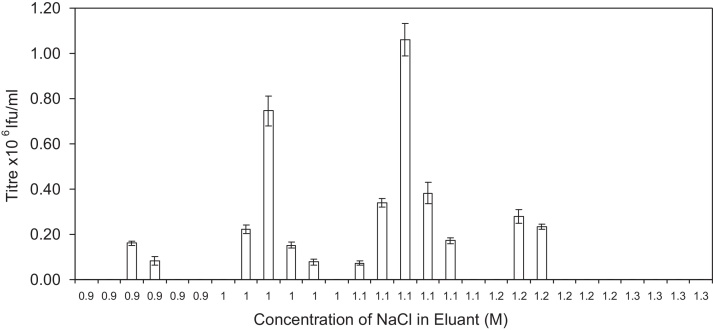
(A) is a bar graph showing the infectious titre of fractions collected during step gradient elution of murine leukaemia virus from a Mustang Q membrane. Load consists of 150 ml of viral supernatant containing 1.71 × 10^7^ ± 3.97 × 10^5^ Ifu, titrated to 0.3 M NaCl 25 mM Tris–HCl pH 8. Gradient elution was from 0.9 M NaCl to 1.5 M NaCl in 100 mM steps. All fractions were 1 ml and all eluants were buffered with 25 mM Tris–HCl. Where no number is reported the viral titre is below the limit of quantitation. Infectivity was measured in triplicate.

In order to establish the maximum attainable purity of γ-retrovirus purified using this Q membrane chromatography device, buffer conditions and its elution profile were analysed. It has been previously established that the application of 0.8 M NaCl pH 8 to the Mustang Q membrane eluted a majority of protein impurities and dsDNA, while not eluting significant quantities of infective virus (data not shown). To identify where MLV elutes from the Mustang Q membrane step gradients between 0.8 M NaCl and 1.5 M NaCl were applied to the membrane utilising steps of 100 mM. Fig. 4 shows that infectious virus was found in multiple fractions at multiple concentrations of NaCl with up to four peaks of elution, ranging from 0.9 M to 1.3 M NaCl, with most of the virus eluting with the application of 1.1 or 1.2 M NaCl 25 mM Tris–HCl pH 8. The occurrence of multiple peaks of elution of infective virus from chromatography matrices has been previously reported; the elution of a lentivirus from a packed bed anion exchange column produced two peaks, which were found to have differing transduction efficiencies [20] and two elution peaks can also be identified from the work published by Kuiper et al. on the use of CHT for virus purification [19].

**Fig. 5:**
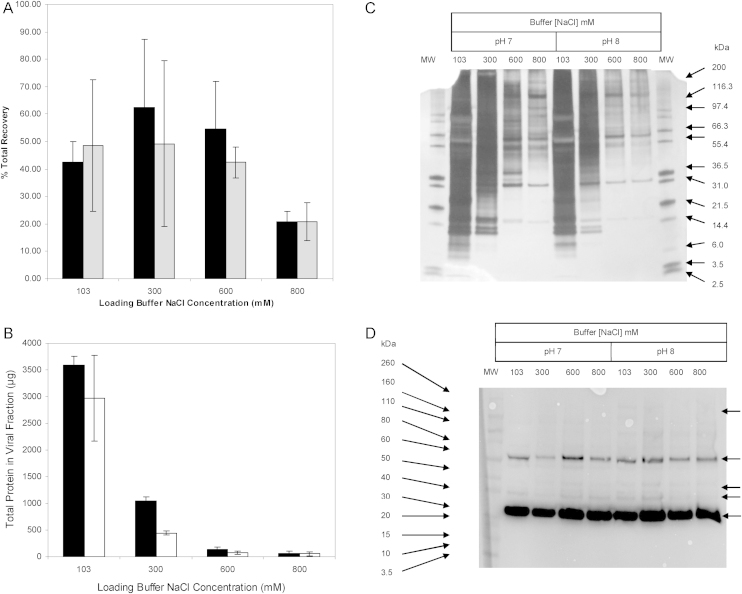
(A) is a bar graph showing the total recovery of infectious virus after binding and elution from Mustang Q membranes of 150 ml samples of murine leukaemia virus containing supernatant adjusted to different NaCl concentrations and pHs. All experiments *n* = 2 except, 103 mM pH 8 NaCl *n* = 11 and 300 mM NaCl pH 8 *n* = 3. (B) is a bar graph that shows the total protein concentration in the infectious virus containing fraction of the eluate of the same experiments, *n* = 2. (C) shows an image of a silver stained SDS-PAGE analysis of infective virus containing elution fractions from one replicate of the above mentioned experiments. (D) shows an image of a western blot of an SDS-PAGE analysis of infective virus containing elution fractions of the above mentioned experiments probed with anti-p30 antibodies. ■ indicates experiments performed at pH 7 and □ indicates experiments performed at pH 8.

To examine the effect of the loading conditions on γ-retrovirus purification, MLV supernatant containing no salt apart from that contained within cell culture media (i.e. approximately 103 mM NaCl), or with additional salt to a concentration of 300, 600, or 800 mM were adjusted to pH 7 ± 0.1 or 8 ± 0.1 using 1 M NaOH and loaded onto a Mustang Q membrane. The membranes were washed and then eluted using 1.3 M NaCl 25 mM Tris–HCl pH 8. Fractions were collected during each step and were assayed for infectious virus and protein content. The total recovery and purity of the eluate from each loading condition was then compared and presented in Fig. 5.

Fig. 5 shows that as the concentration of salt in the load is increased so does the purity of the eluate. The total protein content of the virus containing eluate fractions is greatly reduced with an increase in NaCl concentration and pH, see Fig. 5B. With no additional salt in the load and titration to pH 7 total protein in the eluate was 3592 ± 169 μg, whilst with 800 mM NaCl in the load it was reduced to 57 ± 55 μg. The reduction in total protein was even greater at pH 8, with protein concentration in the eluate of 2968 ± 801 μg and 60 ± 38 μg for a load containing no added NaCl and 800 mM NaCl, respectively. This is over a 100-fold decrease in total protein content compared to the protein content of the load.

Loads titrated to pH 8 provided consistently lower total protein in the eluate when loading at salt concentration below 800 mM compared to pH 7. The increase in purity with increasing loading buffer NaCl concentration or pH is further supported by SDS-PAGE analysis, as shown in Fig. 5C. A range of both high and low molecular weight protein species present in the eluates can be identified. Bands potentially representative of viral capsid protein p30 and matrix protein p15 are present at approximately 30 kDa and 15 kDa. The presence of p30 is confirmed by western blot analysis of the same samples, with strong anti-p30 antibody staining at 30 kDa, see Fig. 5D. This p30 is present as a result of eluted viral particles and potentially as free protein present in the cell culture supernatant.

The presence of fewer and lighter bands in the SDS-PAGE analysis of the eluate fractions processed at higher NaCl concentrations and pH is indicative of the removal of impurities. This increase in purity can be demonstrated in the SDS-PAGE analysis by the presence of a prominent band at around 35 kDa using a load condition of 600 mM NaCl 25 mM Tris–HCl pH 7 and its complete absence using the same conditions at pH 8. The increase in purity with increased concentrations of NaCl can most likely be attributed to increased competition between chloride ions and other anionic species in the load for ion exchange sites on the membrane. This increase in competition for binding will cause impurities with lower affinity for the ion exchange sites to flow through the membrane, with this effect increasing with the concentration of NaCl.

Despite the increase in purity achieved with alteration of the loading buffers, the increase in salt concentration above 600 mM produces an eluate with a reduced average total recovery of infectious virus; this seems especially marked at pH 8. Total recovery of virus varies from between 62.5 ± 24.8% using a load titrated to 300 mM NaCl pH 7 and 20.8 ± 7.8% using a load titrated to 800 mM NaCl. The reduction in recovery with an increase in salt concentration could be caused by many factors, irreversible binding, instability of retrovirus in high salt, or potentially reduced binding in high salt. Low stability of retrovirus in high salt concentration has been previously reported and is therefore a likely factor in loss of infective titre [9,21].

The lower total protein eluted from experiments run with loading buffers at pH 8 is potentially caused by impurities binding irreversibly to the membrane. As mentioned above, such irreversible binding has been observed by Grein and colleagues [17] when assessing different membrane chromatography systems for Baculovirus purification.

**Fig. 6:**
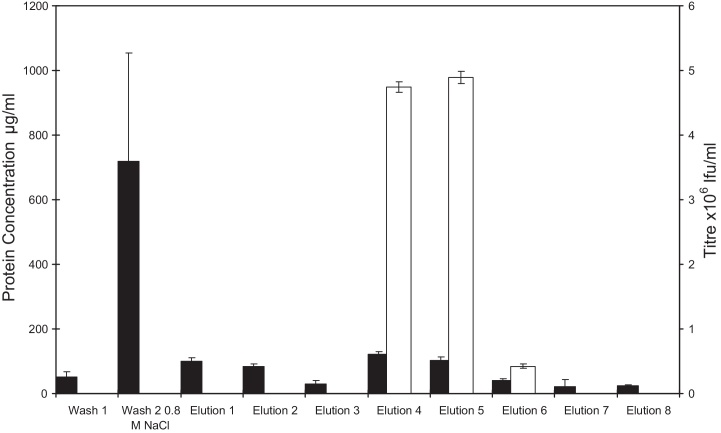
is a bar graph showing the protein concentration and infectious titre of fractions collected during washing and elution of MLV from a Mustang^®^ Q membrane. The load consists of 150 ml of viral supernatant containing 1.33 × 10^7^ ± 8.2 × 10^4^ Ifu, titrated to 0.3 M NaCl 25 mM Tris–HCl pH 8. Washing was performed first with 0.3 M NaCl and then with 0.8 M NaCl. Finally the membrane was eluted with 1.3 M NaCl. All buffers were buffered with 25 mM Tris–HCl pH 8. Where no titre is reported titre is below the limit of detection. ■ indicates protein concentration and □ indicates Ifu.

To examine whether similar final purities to those produced with a high salt load could be achieved using wash buffers of a similarly high salt concentration, experiments utilising 800 mM NaCl washes were performed. Fig. 6 and Table 4 show that addition of an 800 mM NaCl 25 mM Tris–HCl pH 8 wash step into the chromatographic process greatly increases the purity of the eluted virus relative to runs without the high salt wash step. Log removal values of 3.51 and 2.21 are achieved for protein and dsDNA, respectively, when using the extra wash step, which are approximately 1 LRV higher than without it. However, the total protein in the virus containing fractions is higher than that achieved using pH 8 loads containing 600 and 800 mM NaCl or pH 7 800 mM NaCl. The differences in loads, caused by differential dilution to achieve the required load NaCl concentration, could be a factor in the difference between the eluate total protein concentrations of the experiments involving an 800 mM NaCl wash step and 800 mM NaCl concentration in the load. However, the high purities achieved using this additional wash step were also complemented by the relatively high recoveries of 45.3 ± 2.9% and 51.8 ± 5.9% compared to loading in 800 mM NaCl.

The difference in recovery recorded between the high salt loading buffers and the high salt wash buffers indicates that there is a potential detrimental effect on viral infectivity, binding or elution from the membrane that occurs due to loading in high salt. Here, the majority of infectious virus is only recovered from the membrane at salt concentrations above 800 mM NaCl which suggests that the loss of recovery is most likely due to loss of infectivity during application.

The results of these experiments are supported by the data of Rodrigues et al. [22], who found that the optimum wash and elution conditions for a γ-retroviral vector from quaternary amine ion exchange matrices was 864 and 1338 mM NaCl, respectively, buffered with 20 mM phosphate buffer pH 7.5. Using these conditions they were able to provide 1 log of DNA removal and between 2 and 3 logs of removal for protein impurities, results which are matched here with samples 10-fold larger. It is only by using affinity methods such as heparin IMAC that chromatographic methods have produced retroviral vectors of similar purity [6,23,24]. These methods often require chemical or biological modification of the target molecule [25,26], making them unattractive for clinical products. Traditional packed bed chromatographic methods suffer from lower flow rates and therefore increased process time relative to a membrane chromatographic device processing an equivalent volume [22]. While methods such as ultrafiltration have been shown to provide equivalent levels of concentration to those presented here, with Kuiper et al. [19] concentrating a γ-retroviral vector 160 fold using a 100 KDa membrane. However, the level of reduction in impurities such as DNA and protein was minimal.

A further experiment involving a high salt wash step was performed. The resulting eluate was compared to the original MLV containing supernatant using two dimensional differential gel electrophoresis (2D-DiGE). 2D-DiGE allowed the two samples’ protein fingerprint to be compared simultaneously via the pre-staining of each sample with fluorescent dyes. Comparing the pre and post chromatography samples using this method makes it possible to elucidate which protein impurities have had their concentration reduced. Four proteins chosen from the 2D-DiGE were further analysed via mass spectrometry. Of these; one protein was detected only in the feed, Protein 1; one was only detected in the eluate, Protein 2; and two which were detected in both feed and eluate, Protein 3 and 4. A summary of this information can be seen in Table 5.

As shown in Table 5 protein 1 was identified as bovine serum albumin (BSA) and found only in the feed. BSA being present only in the feed demonstrates the removal of protein impurities and highlights the increase in purity produced on elution. Protein 2 identified as the retroviral Capsid protein p30 was detected only in the eluate and once again demonstrates the high concentration factors produced on elution from the Mustang Q membrane. As this protein is too dilute to be detected in the feed but is detected in the eluate sample. Proteins 3 and 4 appear in both the feed and eluate. Therefore, they potentially represent proteins present in the virus, both viral and host cell derived, as well as anionic impurities present in the cell culture media. Protein 3 was determined to be Fetuin, a protein abundant in foetal serum [27]. Therefore, this protein impurity is most likely introduced by the foetal calf serum used as a media additive. Fetuin is present in both load and elution fraction and therefore appears to have a high affinity for the Mustang Q membrane. In order to reduce or remove the burden this protein places on purification, the use of adult sera or the reduction or removal of bovine serum in the cell culture media could be advantageous in providing an eluate with a lower quantity of impurities. Protein 4 was also found in both the load and the eluate and was identified to be heat shock protein 90 (HSP90), although due to the evolutionary conservation of the sequence the peptides mapped identically onto both bovine and human heat shock proteins. Heat shock proteins have previously been found in virus preparations [28–30]. HSP90 is a cytosolic protein used both as a chaperone and for internal signal transduction. Therefore, it is potentially present in the cell culture supernatant and therefore virus preparations due to cell lysis [31]. By reducing the stress on the cells during culture and preventing cell lysis the quantity of this protein released into the supernatant could be reduced. However, it has also recently been established that HSP90 can be found in preparations of both respiratory syncytial virus and HIV-1. In respiratory syncytial virus heat shock proteins have been determined to be important for virus particle formation and maturation [29] while in HIV-1 heat shock proteins have been implicated in viral replication [28]. Therefore, potentially the HSP90 may not be an impurity but a protein involved in virus production.

## 4 Conclusions

Anion exchange membranes have been shown to produce high concentrations of retroviral vector upon elution, up to twice that produced by any chromatography device and over 40 times higher than packed bed devices. Adjusting the concentration of salt or pH in the loading and wash buffers makes it possible to produce an eluate of high purity. The use of a high salt wash buffer provides for greater recovery than the use of a loading buffer of equivalent salt concentration, whilst producing similar levels of impurity removal, thus indicating a time dependant inactivation of virus in the presence of high concentrations of NaCl. Using this system of binding the γ-retrovirus to the Mustang Q membrane followed by a high salt wash allowed for LRVs of 3.51 for protein and 2.21 for DNA. The primary protein impurity removed by this wash step was identified by mass spectrometry as being BSA. It was also established that some proteins such as Fetuin and HSP90 were co-purified with viral proteins and therefore alternative methods of removal are required for them, potentially by reducing or removing them from cell culture media. Additionally, it was determined that the Mustang Q membrane was able to adsorb very large loads of viral supernatant, equivalent to 2857-fold larger than the Q membrane volume. The capacity of the Mustang Q membrane for γ-retroviruses was also determined as at least 1.27 × 10^8^ Ifu/ml of membrane, the first report of a chromatographic membrane's capacity for a γ-retrovirus.

## Figures and Tables

**Table 1:** shows selected methods of virus purification and concentration and their ability to concentrate retroviruses.

Device	Virus	Concentration factor	Reference
Streptavidin Magnespheres^®^	Lentivirus	2500	[18]
Mustang^®^ 0.18 ml	Lentivirus	∼140 Estimate	[8]
Fractogel^®^ DEAE	γ-Retrovirus	1.25–5	[14]
Ultrafiltration 100 kDa	γ-Retrovirus	160	[19]
LentiSELECT 500	γ-Retrovirus	11	[9]

**Table 2:** shows the total load, flow through, recovery and concentration factor achieved when murine leukaemia based viral vectors are bound to and eluted from the Mustang Q coin unit.

Load (Ifu)	Load (ml)	Flow through (%)	Recovery (Ifu) recovery (%)		Peak concentration factor (fold)	Total concentration factor (fold)
9.29 × 10^7^ ± 5.25 × 10^6^	800	29.1 ± 1.4	3.9 × 10^7^ ± 5.99 × 10^6^	44.8 ± 6.9	404	125
7.72 × 10^7^ ± 2.01 × 10^5^	790	<30	4.11 × 10^7^ ± 5.65 × 10^6^	56.7 ± 3.0	420	140
7.86 × 10^7^ ± 3.36 × 10^6^	1000	n/a	3.25 × 10^7^ ± 2.74 × 10^6^	44.1 ± 3.7	321	132

**Table 3:** Shows the total load, flow through, recovery and concentration factor achieved when 1775 ml of murine leukaemia based viral vector containing supernatant titrated to 300 mM NaCl 25 mM Tris–HCl pH 7 is bound to and eluted from the Mustang Q XT Acrodisc.

	Load	Load concentration	Flow through	Recovery	Recovery (%)	Peak concentration factor	Total concentration factor
Infectious virus	2.39 × 10^8^ ± 3.77 × 10^5^ Ifu	1.26 × 10^5^ ± 8.68 × 10^3^ Ifu	n/a	1.08 × 10^8^ ± 1.61 × 10^7^ Ifu	48.29 ± 7.18	410	179
p30 Protein	1.04 × 10^5^ ± 15.5 ng	64.46 ± 0.38 ng/ml	5.32 × 10^4^ ± 11.1 ng	2.34 × 10^4^ ± 24.4 ng	22.47 ± 0.01	n/a	30

**Table 4:** Shows the results of purification runs of murine leukaemia virus using a Mustang® Q anion exchange membrane utilising different buffer conditions.

	No added salt in load	300 mM NaCl in load	0.8 M Wash 1	0.8 M Wash 2	0.8 M Wash combined
Recovery (%)	48.5 ± 23.9 (n = 11)	49.3 ± 30.3 (n = 3)	45.3 ± 2.9	51.8 ± 5.9	48.5 ± 8.0
Total dsDNA in load (mg)	13.3 ± 6.88	9.5 ± 0.06	n/a	10.01 ± 0.01	n/a
Total dsDNA in eluate post RNase (mg)	0.378 ± 0.005	0.146 ± 0.005	0.029 ± 0.004	0.060 ± 0.004	0.045 ± 0.022
LRV for DNA	1.55	1.81	n/a	2.21	n/a
Total protein in eluate (μg)	2968.4 ± 801.6	445.9 ± 42.6	160.3 ± 9.8	88.0 ± 44.8	124.2 ± 68.6
LRV for protein	2.18 ± 0.20	2.60 ± 0.55	3.34	3.68	3.51 ± 0.24
Average increase in specific titre (fold)	104 ± 48	557 ± 34	1257	1122	1189 ± 95

**Table 5:** shows a summary of the proteins identified by electrospray mass spectrometry after 2D-DiGE of Pre and Post Chromatography MLV samples.

Protein number	Found in	Identified as
1	Feed	BSA
2	Eluate	p30 Capsid protein
3	Feed and eluate	alpha-2-HS-glycoprotein ‘Fetuin’
4	Feed and eluate	HSP 90
